# Evaluation of normal findings using a detailed and focused technique for transcutaneous abdominal ultrasonography in the horse

**DOI:** 10.1186/1746-6148-10-S1-S5

**Published:** 2014-07-07

**Authors:** Sarah Williams, Jonathan David Cooper, Sarah Louise Freeman

**Affiliations:** 1 Dr S. Williams’ current address is 47 Ashby Grove, Loughborough, Leicestershire, UK. LE11 3AP; 2 Mr. Cooper’s current address is The Minster Veterinary Practice, Salisbury Rd, York. YO26 4YN; 3 School of Veterinary Medicine and Science, University of Nottingham, College Road, Sutton Bonington, Loughborough, Leicestershire. LE12 5RD

## Abstract

**Background:**

Ultrasonography is an important diagnostic tool in the investigation of abdominal disease in the horse. Several factors may affect the ability to image different structures within the abdomen. The aim of the study was to describe the repeatability of identification of abdominal structures in normal horses using a detailed ultrasonographic examination technique and using a focused, limited preparation technique.

**Methods:**

A detailed abdominal ultrasound examination was performed in five normal horses, repeated on five occasions (total of 25 examinations). The abdomen was divided into ten different imaging sites, and structures identified in each site were recorded. Five imaging sites were then selected for a single focused ultrasound examination in 20 normal horses. Limited patient preparation was performed. Structures were recorded as ‘identified’ if ultrasonographic features could be distinguished. The location of organs and their frequency of identification were recorded. Data from both phases were analysed to determine repeatability of identification of structures in each examination (irrespective of imaging site), and for each imaging site.

**Results:**

Caecum, colon, spleen, liver and right kidney were repeatably identified using the detailed technique, and had defined locations. Large colon and right kidney were identified in 100% of examinations with both techniques. Liver, spleen, caecum, duodenum and other small intestine were identified more frequently with the detailed examination. Small intestine was most frequently identified in the ventral abdomen, its identification varied markedly within and between horses, and required repeated examinations in some horses. Left kidney could not be identified in every horse using either technique. Sacculated colon was identified in all ventral sites, and was infrequently identified in dorsal sites.

**Conclusions:**

Caecum, sacculated large intestine, spleen, liver and right kidney were consistently identified with both techniques. There were some normal variations which should be considered when interpreting ultrasonographic findings in clinical cases: left kidney was not always identified, sacculated colon was occasionally identified in dorsal flank sites. Multiple imaging sites and repeated examinations may be required to identify small intestine. A focused examination identified most key structures, but has some limitations compared to a detailed examination.

## Background

Transcutaneous abdominal ultrasonography is used for the investigation of acute and chronic abdominal disorders [1-4]. As with all imaging tools, there are limitations to the technique. In the horse, key limitations are the size and depth of the abdomen, the mobility of some abdominal structures, and acoustic shadowing from the lungs and large intestine. Previous studies have reported the normal location and appearance of different abdominal organs [4-9], but only one study [8] evaluated how consistently structures may be identified. Interpretation of pathologic change requires an understanding of expected ‘normal’ findings based on breed and species.

A detailed abdominal ultrasound examination may not be possible in some clinical situations, due to the clinical status of the patient. In these cases, a shorter duration technique which involves limited patient preparation (not clipping the coat hair), and ‘focusing’ the examination on selected sites of the abdomen may be appropriate [4,10]. In human medicine ‘focused examinations’ are commonly used in the emergency assessment of abdominal disease. Focused examinations are often employed ‘to answer a single question’, such as the presence or absence of peritoneal fluid in trauma patients [11]. The use of focused examinations in equine patients with gastrointestinal disease has been reported [4,10], but normal findings, and comparison to a detailed examination has not been described.

The study was divided into two phases. The aim of the first phase was to document the repeatability of findings using a detailed (whole abdomen) transcutaneous ultrasonographic examination in horses following clipping of the coat hair. The aims of the second phase were to describe the normal repeatability of findings using a focused transcutaneous ultrasonographic examination (selected imaging sites), with limited patient preparation, and to compare the findings with the detailed examination technique.

The study objectives were:

i. To document which abdominal structures could be repeatedly identified using a detailed transcutaneous ultrasonography technique and a focused examination technique

ii. To determine where different abdominal structures could be identified, and identify imaging sites for a ‘focused’ examination

iii. To describe any variation that occurred within and between different horses

iv. To compare the repeatability of findings between a detailed and a focused examination technique

## Methods

The study was reviewed and approved by the School of Veterinary Medicine and Science Ethics Committee.

### Study population

The study was performed in horses belonging to the Defence Animal Centre, Melton Mowbray. The five horses for the first phase (detailed examination) were hospitalized for musculoskeletal disorders; all were stabled and either maintained on box rest or limited sand paddock turn out. The 20 horses used in the second phase (focused examination) were working horses, stabled full time, and on a standardized exercise regime. Both groups were maintained under standardized feeding and health care regimes, and inclusion criteria were that horses were free from existing known gastrointestinal disease. The horses were restrained in stocks or a stable with a headcollar only, and no other physical or chemical restraint was used. The examinations were performed at similar times of day (between 0900h and 1200h, and between 1300h and 1600h, and the horses were fed twice daily at 0700h and 1630h).

### Detailed examination technique

A detailed transcutaneous abdominal ultrasound examination was performed in five normal horses. The abdomen was divided into ten different imaging sites, and the structures identified in each site were recorded. The examination was repeated in each animal on five separate occasions within a 14 day period. The duration of each examination, including preparation, was recorded.

Each horse was prepared by surgically clipping the abdomen, cleansing with antiseptic solution (Hibiscrub)*^1^ and alcohol (Surgical Spirit)*^2^, followed by application of a coupling gel (BCF Technology Ltd)*^3^. Ultrasonographic examination was performed using a MyLab 30 with a 3.5MHz sector scanner (Esaote)*^4^, which has a maximum depth of penetration of 36 cm. The depth was initially set to the maximum penetration to identify the structures present, then adjusted to different depths according to the individual structure being examined.

**Figure 1:**
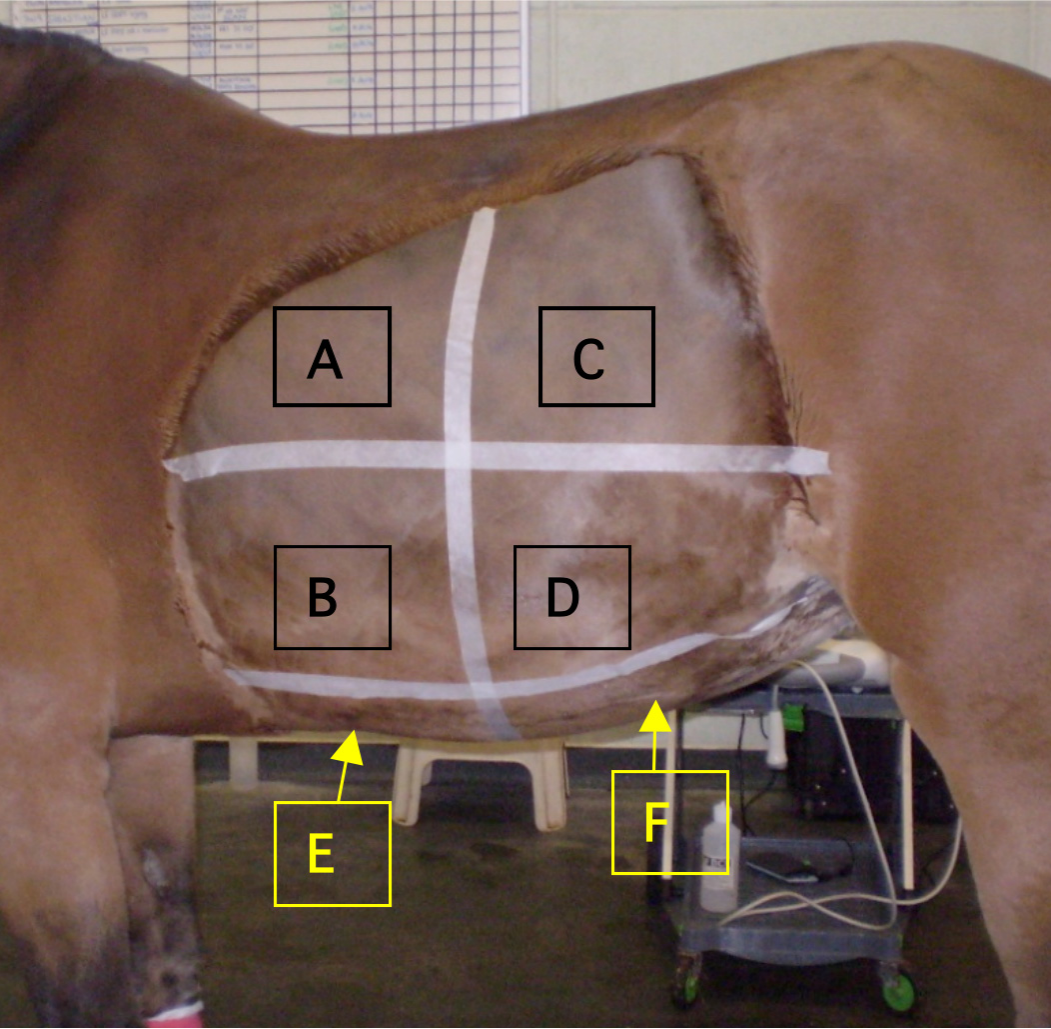
**Photograph of the left abdomen illustrating the six regions for ultrasonographic examination.** Sites A, B, C and D were repeated on the right abdomen. **A:** Left craniodorsal flank, **B:** Left cranioventral flank, **C:** Left caudodorsal flank, **D:** Left caudoventral flank , **E:** Cranioventral, **F:** Caudoventral

The abdomen was divided into ten different imaging sites, (four on each flank and two in the ventral abdomen), using anatomical landmarks. Flank sites were defined dorsally as a curved line extending from the tuber coxae along the sublumbar musculature and lung line to the 6^th^ intercostal space, and ventrally as a horizontal line from the olecranon to the stifle joint. Each flank was bisected craniocaudally and dorsoventrally, creating four sites - craniodorsal, cranioventral, caudodorsal and caudoventral flank (Figure 1).

The ventral abdomen was defined as ventral to the flank sites, extending from the sternum cranially to the pelvis caudally. It was divided into cranioventral and caudoventral sites by continuing the lines that bisected the flanks (Figure 1).

The flank sites were imaged by taking sequential images in the transverse plane across each site, moving the transducer continuously from dorsal to ventral, starting in the most cranial aspect, then repositioning the transducer approximately 5cm caudal, and repeating the dorsal to ventral sweep of transverse images. The ventral abdomen was imaged by taking sequential images in the sagittal plane across each site, moving the transducer continuously from cranial to caudal, starting at the midline, then repositioning the transducer approximately 5cm lateral and repeating the cranial to caudal sweep of sagittal images.

**Figure 2:**
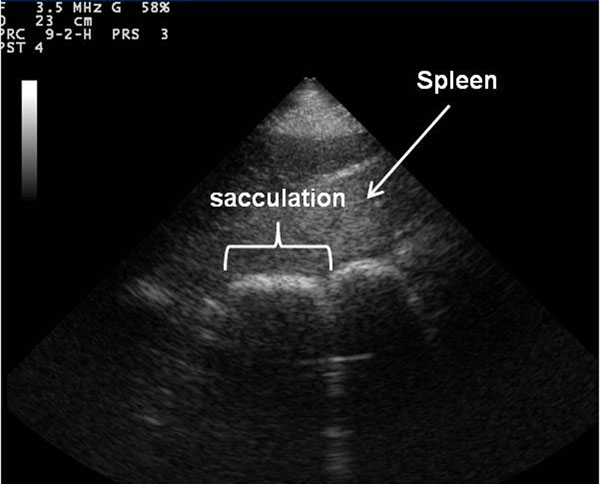
**Ultrasonographic image of sacculated large intestine.** Frontal plane ultrasound image of sacculated large intestine with cranial to the left of the image obtained in the left ventral region of the abdomen using a detailed transcutaneous ultrasound technique.

The following abdominal structures were identified as being present or absent for each site: kidney, liver, spleen, duodenum, small intestine, caecum, sacculated colon and non-sacculated colon. Abdominal organs were only recorded as present if distinguishing features could be identified on the ultrasonographic images, e.g. the cortex and the medulla for the kidney [5,6]. The stomach was not evaluated as there were inconsistencies in identifying this organ during pilot work. Small intestine was recorded as ‘duodenum’ if identified in the typical location for duodenum [8], and ‘small intestine’ if identified at other sites. Small intestine was identified by its small diameter, and semi-fluid contents. Large intestine was recorded as caecum, sacculated colon or non-sacculated colon. Large intestine was identified by its large diameter, and by the acoustic shadowing from the contents of the lumen. Caecum was distinguished by its location, contents and orientation of contractions. It was differentiated from ventral colon in the right abdomen by following it to its base in the caudodorsal abdomen. Distinguishing which part of the large colon (left dorsal colon, left ventral colon etc.) is being imaged requires either direct visualisation or palpation of anatomical features. Therefore large intestine was recorded as sacculated colon if sacculations or indentations of the wall were identified (Figure 2). It was recorded as non-sacculated colon if there were no visible sacculations.

### Focused examination technique

Based on the results of the first phase, five sites were selected to image key abdominal organs (kidneys, spleen, liver, small intestine, caecum and large intestine) and common sites of disease (such as renal and hepatic disease, large colon displacements and torsions, and epiploic foramen entrapments). These sites were: left caudodorsal flank (best imaging site for left kidney and spleen, disease site for nephrosplenic entrapments), right caudodorsal flank (best imaging site for duodenum, right kidney and caecum), right craniodorsal flank (best imaging site for non-sacculated large intestine and liver), cranioventral and caudoventral abdomen (imaging sites for ventral colon, and for diseased small intestine). Sites were defined as previously described. The focused examination was performed on a single occasion in 20 normal horses, using minimal preparation. The horses were examined in November, and all had had a coarse body clip for work. No further clipping was performed, and the sites were prepared by applying alcohol, followed by coupling gel. The identification of structures and duration of examination were recorded as previously described.

All examinations were performed by two authors (SW and JC), and images were recorded and stored electronically for review. Training for both operators (by SF) included principles and physics of ultrasonography, practical training on equipment set up and use, canine and equine abdominal anatomy and ultrasonographic technique, supervision of examinations, and review of selected images.

### Data analysis

Data from each phase was analysed by tabulating where an abdominal structure was identified on each examination. The ability to repeatedly identify abdominal structures was determined by calculating:

• How often a structure was identified in each imaging site (expressed as a % of the total number of examinations)

• How often a structure was identified in each examination, irrespective of imaging site (expressed as a % of the total number of examinations)

• Where each structure was most repeatability identified (termed ‘best imaging site’)

For the detailed examination technique, if a structure was identified on >1 and <24 examinations, the data was further analysed to determine whether the repeatability varied within or between individual animals, by tabulating the numbers of times and locations that it was recorded for each horse on each examination and comparing these within and between animals.

## Results

### Detailed examination technique

The horses were aged 11.6 +/- 3.4 years, with a body weight of 586 +/-35.9kg, and a body condition score of 3.6 +/- 0.5 (mean +/- SD). They were all Irish draft or Irish draft cross, and height ranged from 15.2-16.1hh.

The location, repeatability and best imaging site for each abdominal structure is shown in Table 1. Duodenum was not identified on at least one examination in each horse, but was identified in every horse across all five examinations. The location and number of identification sites for small intestine varied both between different horses and within repeated examinations on the same horses (Table 2). The ultrasonographic features of the left kidney could not be identified on three examinations, all of which occurred in the same horse. The urinary bladder was only identified in two horses on one examination each (2/25 (8%)) in the caudoventral abdomen imaging site.

**Table 1:** Identification sites and repeatability of identification (%) of abdominal structures, imaged using a detailed transcutaneous ultrasonographic technique in five normal horses, repeated on five occasions (total of 25 examinations).

	Repeatability of identification (%) at each individual imaging site*										
Abdominal structure	Right caudo-dorsal flank	Right caudo-ventral flank	Right cranio-dorsal flank	Right cranio- ventral flank	Left caudo-dorsal flank	Left caudo-ventral flank	Left cranio-dorsal flank	Left cranio- ventral flank	Cranio-ventral abdomen	Caudo-ventral abdomen	Repeatability of identification across complete examination (examinations identified / total number of examinations)**

Duodenum	**72**	0	0	0	0	0	0	0	0	0	72% (18/25)

Other small intestine	0	**40**	32	28	12	8	36	**40**	36	32	76% (19/25)

Caecum	**100**	88	0	0	0	0	0	0	0	0	100% (25/25)

Sacculated large intestine	56	95	12	**100**	**100**	**100**	84	**100**	**100**	**100**	100% (25/25)

Non-sacculated large intestine	32	4	**88**	0	12	0	44	0	0	0	92% (23/25)

Right kidney	**100**	0	0	0	0	0	0	0	0	0	100% (25/25)

Left kidney	0	0	0	0	**88**	0	0	0	0	0	88% (22/25)

Liver	0	0	**100**	80	0	0	0	16	0	0	100% (25/25)

Spleen	0	0	0	0	**100**	**100**	**100**	**100**	32	56	100% (25/25)

*The repeatability of identification (%) at each individual imaging site describes how often each abdominal structure was identified at each separate imaging site (across all 25 examinations (five horses examined five times)), i.e. where can you find this structure on ultrasonographic examination?

** The repeatability of identification across complete examination describes how often each abdominal structure was identified at any site during each examination (total of 25 examinations (five horses examined five times)), i.e. how often can you find this structure on ultrasonographic examination of the whole abdomen?

The site where each structure was most repeatedly identified, termed the best imaging site is highlighted in bold.

**Table 2:** Number of different abdominal locations (out of a total 10 regions) that the small intestine (excluding duodenum) was identified using a detailed transcutaneous ultrasonographic examination in 5 normal horses, repeated on 5 different occasions.

	1^st^ ultrasound examination	2^nd^ ultrasound examination	3^rd^ ultrasound examination	4^th^ ultrasound examination	5^th^ ultrasound examination
Horse 1	1 location	8 locations	1 location	not identified	1 location

Horse 2	1 location	2 locations	1 location	3 locations	1 location

Horse 3	not identified	4 locations	3 locations	5 locations	2 locations

Horse 4	3 locations	2 locations	not identified	not identified	4 locations

Horse 5	3 locations	2 locations	2 locations	2 locations	2 locations

Number of locations that small intestine was identified on each examination

### Focused examination technique

The horses were aged 8.8 +/-2.9 years (mean +/- SD), height ranged from 16.1-17.3hh, and breed was either Irish Draft cross, or Warmbloods. Bodyweight and body condition scores were not obtained in this population.

Sacculated large intestine and right kidney were identified in every examination, other structures were identified less frequently (Table 3). The duodenum, small intestine, caecum, spleen and were identified less frequently compared to the detailed examination (Table 3). Urinary bladder and non-sacculated colon were not identified with the focused technique.

**Table 3:** Identification sites and repeatability of identification (%) of abdominal structures, imaged using a single focused ultrasound examination in 20 normal horses (total of 20 examinations).

	Repeatability of identification (%) at each individual imaging site*					
Abdominal structure	Right caudo-dorsal flank	Right cranio- dorsal flank	Left caudo-dorsal flank	Cranio-ventral abdomen	Caudo-ventral abdomen	Repeatability of identification of structure across complete examination (examinations identified / total number of examinations)**

Duodenum	**30**	0	0	0	0	30% (6/20)

Other small intestine	0	0	0	**35**	10.5	45.5% (9/20)

Caecum	**85**	0	0	0	0	85% (17/20)

Sacculated large intestine	15	**100**	90	**100**	**100**	100% (20/20)

Right kidney	**100**	0	0	0	0	100% (20/20)

Left kidney	0	0	**90**	0	0	90% (18/20)

Liver	0	**95**	0	0	0	95% (19/20)

Spleen	0	0	**80**	0	0	80% (16/20)

*The repeatability of identification (%) at each individual imaging site describes how often each abdominal structure was identified at each separate imaging site (across all 20 examinations (20 horses examined once)), i.e. where can you find this structure on ultrasonographic examination?

** The repeatability of identification across complete examination describes how often each abdominal structure was identified at any site during each examination (total of 20 examinations (20 horses examined once)), i.e. how often can you find this structure on ultrasonographic examination of the whole abdomen?

The site where each structure was most repeatedly identified, termed the ‘best imaging site’ is highlighted in bold.

The best imaging sites for duodenum, caecum, sacculated large intestine, right and left kidney, liver and spleen were the same as for the detailed technique. Small intestine was only identified in the ventral abdomen, and the cranioventral site was the best imaging site (Table 3).

### Duration of techniques

Clipping and preparation for the detailed systematic examination took 45 minutes, and the ultrasound took between 60 -120 minutes. The duration of the focused technique was 20 minutes, including preparation.

## Discussion

Ultrasound is a valuable diagnostic technique, but has some limitations related to its physical properties. The large difference in acoustic impedances between soft tissues and bone or air results in reflection of most of the diagnostic ultrasound waves at these interfaces and results in shadowing [12]. This acoustic shadowing prevents imaging of deeper structures. In practical terms, this means that the lungs and gas filled stomach limit the acoustic window in the cranial thorax, and the extensive large colon in the horse causing acoustic shadowing from its contents throughout the abdomen [13], these were the main factors interfering with imaging in this study. Acoustic shadowing from the thorax mainly affects craniodorsal structures, such as the liver. The right lobe of the equine liver extends further caudally than the left [14], and in this study, liver was identified frequently on the right side, but rarely on the left side. The equine large colon lies along the ventral and flank body walls, and acoustic shadowing from colonic gas can affect large areas of the abdomen. This was a significant problem, even in the normal horses in this study, and probably contributed to the low frequency of identifying structures such as small intestine and urinary bladder. Ability to visualise the bladder will also depend on the imaging window and the degree of filling present. Acoustic shadowing is likely to be more marked in horses with gaseous distension of the large colon.

Limited site preparation and body wall thickness both cause scatter and reflection of ultrasound causing attenuation of the ultrasound beam and reduced image quality. As expected, structures were identified more frequently with the detailed technique where site preparation involved clipping and scrubbing. Body wall thickness varies depending on the region of the abdomen and may explain some of the differences between sites. Muscle layers are thinnest ventrally, where only one muscle is present, compared to the caudodorsal abdomen which has three layers of abdominal muscles [14]. The combined factors of limited preparation and increased body wall thickness probably explain the reduced identification of organs in the caudodorsal flank sites with the focused technique: caecum, liver and spleen were identified less frequently compared to the detailed technique, and small intestine was not identified in any flank sites with the focused technique. The depth of a structure and any overlying organs also affects ultrasound attenuation, and this may explain why the left kidney was identified less frequently than the right kidney. The left kidney lies beneath the spleen and is deeper within the abdomen [5]. In this study, the right kidney was identified in every horse, but the left kidney was only positively identified on 22/25 detailed examinations and 18/20 focused examinations. In this study, a structure was recorded as identified if anatomical features could be distinguished (i.e. images were of diagnostic quality). The decreased frequency of identification of the spleen and kidney may be explained by the limited preparation and not clipping with the focused technique, resulting in interference and poorer quality images in the near field. The frequency of identification of the kidney may also be improved by using different imaging windows, including a more dorsal approach to this structure. Busoni et al. (2010) reported not being able to image the left kidney in seven of 36 horses with abdominal pain, only two of which had a nephrosplenic entrapment. One of the ultrasonographic features of nephrosplenic entrapment is being unable to visualise the left kidney [3,5]. The current study shows that using several ultrasonographic features, as described by Santschi et al (1993), is important to avoid a false positive identification for this condition.

Epstein et al. (2008) describes the identification and characteristics of normal stomach, duodenum, jejunum, caecum, and peritoneal fluid. Conversely to the current study, Epstein et al. identified duodenum and jejunum in all nine normal ponies in the study. However, size difference in current study versus Epstein et al. (2008), where ponies weighing 108-216kg were used likely accounts for the lower frequency of identification of structures described herein. It was also only identified in the right caudodorsal flank in this study, which is also the site described by Bithell et al. (2010), but it can also be imaged in the right craniodorsal flank deep to the liver [8].

Small intestine (jejunum or ileum) was highly variable in how frequently and where it was identified. With the detailed technique, it was identified at every location except the right caudodorsal abdomen, but the ventral sites were most consistent. This was consistent with the study by Epstein et al. (2008), which identified jejunum at multiple different locations, and reported the highest frequency at left dorsal and ventral locations. These differences again may relate to differences in horse size or in diet or other factors affecting the acoustic windows and imaging quality. The variation in sites is likely to be due to the mobility of the small intestine within the abdomen [14], rather than a feature of an individual animal. In one horse, for example, small intestine could not be identified at any location on one examination, (despite altering the depth of the imaging window) but was identified at eight locations on a subsequent examination. Repeated examinations may therefore be of value in animals with suspected small intestinal disease.

Sacculated large intestine was the main structure identified using both techniques. It was identified consistently at all ventral sites, but was also identified at some dorsal sites. Hendrickson et al. (2007) also reported imaging sacculations in both dorsal and ventral sites, and attributed this to peristaltic activity causing segmentation of non-sacculated dorsal colon. However, there are other possible explanations. There is some debate on the presence of sacculations in the dorsal colon within the anatomy literature with Dyce et al. (2002) describing a return of sacculations within the dorsal colon, but other authors describing this region as non-sacculated. The most appropriate description is probably Koenig and Liebig (2007) who describe the diaphragmatic flexure and right dorsal colon as having relatively indistinct sacculations [15]. Anatomical examination of this region shows that there are some sacculations in this region, but these are wider and less pronounced than in the ventral colon. There are therefore two possible explanations for the presence of sacculated colon within dorsal sites – this may relate to the indistinct sacculations which start to reappear through the right dorsal colon. In this study, non sacculated colon was identified in the right dorsal site, with the frequency reducing from cranial to caudal, as expected from the anatomical return of sacculations. The sacculated colon may also have been due to imaging of other regions of the colon, for example, small colon, which is located in the left caudodorsal abdomen, or left ventral colon, which is much larger in diameter than the left dorsal colon [14], so may extend into dorsal regions.

In one of the 25 detailed examinations (4% frequency), non-sacculated colon was identified in a ventral flank site; this may have been diaphragmatic flexure, and therefore relates to the degree of distension and location of different regions of the colon, or the designation of sites as ventral and dorsal. Sacculated colon was identified more frequently in the right craniodorsal flank region using the focused examination technique – this may have been due to differences in the diet and management of the hospitalised horses in the detailed examination, vs the horses in work used for the focused examination, or again be related to the poorer imaging quality associated with the limited preparation of the focused technique which may have reduced the accuracy to distinguish between sacculated and non-sacculated colon in a region where the sacculations are indistinct. Distinguishing which part of the colon is being imaged requires either direct visualisation or palpation of anatomical features, which was not possible in the current study. Therefore the authors elected to describe colon as either sacculated or non-sacculated. There is clearly some debate as to the degree of demarcation and how these should be defined. As defined in this study, sacculated colon on the left side could therefore have been either left ventral colon or small colon. Sacculated colon on the right side would include both right ventral colon, small colon and possibly right dorsal colon.

Focused ultrasonographic techniques have been described in the evaluation of colic patients [4,10]. We selected five sites based on the findings of the detailed examination, and the main areas of interest (small intestinal disease, large intestinal displacement and impaction, hepatic and renal disease). Busoni et al. (2010) used seven imaging sites, which have some overlap with sites used in this study. Both Klohnen et al. (1996) and Busoni et al. (2010) performed transcutaneous ultrasonography without clipping [4,10]. In this study, limited preparation did affect image quality, and identification of structures was reduced compared to the detailed examination, particularly for normal small intestine, which will be an important consideration when using different techniques in clinical cases. Differences between the two techniques were not evaluated by statistical analysis, as the nature of the data and the numbers of animals involved meant that descriptive analysis was most appropriate. Busoni et al. (2010) description of the FLASH technique in 36 horses concentrated on ease and value of technique, and on abnormal findings in specific structures. The duration of their examination was shorter (mean 10.7 minutes, range 7 - 17) and fewer organs were assessed compared to this study. Emergency focused assessment with sonography (FAST) in humans is short duration (5-10 minutes), but it is focused on answering a single clinical question, not assessing multiple organs [11]. Assessment of colic patients may therefore require a longer examination, or targeting of selected sites based on the clinical question. The findings of this study should assist clinicians to select the optimal imaging sites for different abdominal organs, and in deciding whether a detailed or focused examination technique is most appropriate.

## Conclusions

This study describes the normal ultrasonographic findings using a detailed and a focused ultrasonographic examination technique. Caecum, sacculated large intestine, spleen, liver and right kidney were consistent landmarks that would be expected to be identified during abdominal examinations. Normal variations should be considered when interpreting ultrasonographic findings in clinical cases such as: difficulty imaging left kidney in some animals, sacculated colon occasionally identified in dorsal flank sites, and that the location of small intestine can be variable and require repeated examinations before positive identification in some horses. The duration of the focused technique was 20 minutes, including preparation. Most structures were identified with the focused technique, but with a lower frequency compared to the detailed technique, especially in caudodorsal flank sites, and small intestine was only identified in the ventral abdomen.

This study provides data to help interpret findings on abdominal ultrasonography in the horse, and to assist clinicians in selecting appropriate sites for focused examinations of different abdominal organs.

## Competing interests

The authors have no competing interests.

## Authors' contributions

SW and JC performed all ultrasound examinations. SF, SW and JC all contributed to study design. SF provided training in techniques and supervision. SW and SF were primary authors of the manuscript, but all authors contributed to the final version.

## Declarations

Open access publication of this research was funded by the Eleventh Equine Colic Research Symposium.

This article has been published as part of *BMC Veterinary Research* Volume 10 Supplement 1, 2014: Selected articles from the Eleventh International Equine Colic Research Symposium. The full contents of the supplement are available online at http://www.biomedcentral.com/bmcvetres/supplements/10/S1. Publication of this supplement has been funded by The British Equine Veterinary Association.

## Manufacturers addresses

*^1^Hibiscrub, Regent Medical Ltd, Bedfordshire, UK

*^2^Surgical Spirit, Battle, Hayward and Bower Limited, Lincoln, UK

*^3^BCF Technology Ltd, Livingstone, UK

*^4^Esaote Group, Berkshire, UK
